# Incommensurate Phase in Λ‐cobalt (III) Sepulchrate Trinitrate Governed by Highly Competitive N−H⋅⋅⋅O and C−H⋅⋅⋅O Hydrogen Bond Networks**

**DOI:** 10.1002/chem.202104151

**Published:** 2022-02-10

**Authors:** Somnath Dey, Andreas Schönleber, Sander van Smaalen, Wolfgang Morgenroth, Finn Krebs Larsen

**Affiliations:** ^1^ Laboratory of Crystallography University of Bayreuth Universitätsstraße 30 95447 Bayreuth Germany; ^2^ Institute of Crystallography RWTH Aachen University Jägerstraße 17–19 52066 Aachen Germany; ^3^ Institute of Geosciences University of Potsdam Karl-Liebknecht-Straße 24–25 14476 Potsdam-Golm Germany; ^4^ Department of Chemistry Aarhus University Langelandsgade 140 8000 Aarhus C Denmark

**Keywords:** hydrogen bond, incommensurate modulation, phase transition, steric factors, twin domains

## Abstract

Phase transitions in molecular crystals are often determined by intermolecular interactions. The cage complex of [Co(C_12_H_30_N_8_)]^3+^ ⋅ 3 NO_3_^−^ is reported to undergo a disorder‐order phase transition at *T*_c1_ ≈133 K upon cooling. Temperature‐dependent neutron and synchrotron diffraction experiments revealed satellite reflections in addition to main reflections in the diffraction patterns below *T*_c1_. The modulation wave vector varies as function of temperature and locks in at *T*_c3_≈98 K. Here, we demonstrate that the crystal symmetry lowers from hexagonal to monoclinic in the incommensurately modulated phases in *T*_c1_<*T*<*T*_c3_. Distinctive levels of competitions: trade‐off between longer N−H⋅⋅⋅O and shorter C−H⋅⋅⋅O hydrogen bonds; steric constraints to dense C−H⋅⋅⋅O bonds give rise to pronounced modulation of the basic structure. Severely frustrated crystal packing in the incommensurate phase is precursor to optimal balance of intermolecular interactions in the lock‐in phase.

## Graphical abstract

**The intermediate incommensurate modulated structure** of Λ‐cobalt (III) sepulchrate trinitrate is ordered yet retains amplitude of molecular rotations common to high temperature disordered as well as low temperature high *Z*′ structure. Contributions from bifurcated N−H⋅⋅⋅O bonds as well as very short H⋅⋅⋅H contacts in addition to C−H⋅⋅⋅O bonds leads to severe frustrations in crystal packing than that in the lock‐in phase.

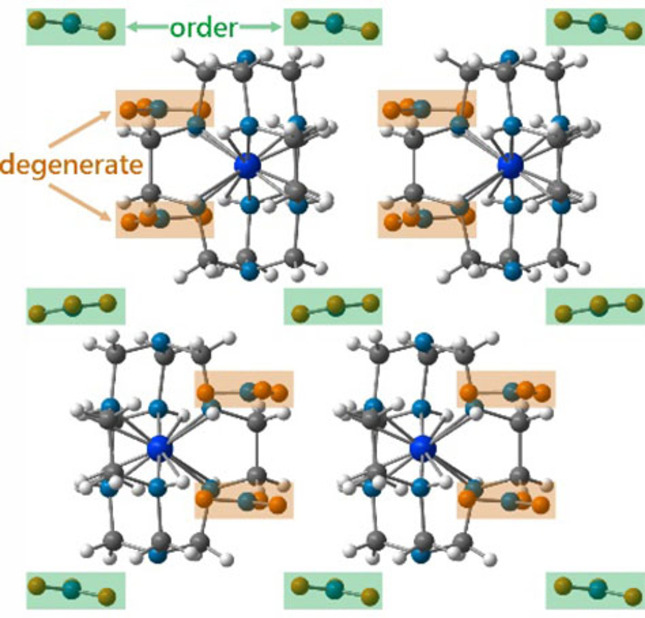

## Introduction

In the field of molecular crystals non‐covalent interactions (namely, hydrogen bonds, halogen bonds, halogen⋅⋅⋅halogen interactions, van der Waals interactions) play an important role for the crystal packing and the supramolecular assemblies.^[1]^ These interactions determine properties like conductivity, piezoelectricity and ferroelectricity, as well as optical and catalytic properties.^[2]^ Their function, stability and balance in crystal packing and in phase transitions were studied by various theoretical and experimental techniques as function of temperature and pressure (especially hydrogen bonds).^[3]^

Incommensurately and commensurately modulated molecular crystal structures often originate in the competition between intra‐ and intermolecular forces or between different intermolecular interactions. Sometimes they mimic the molecular conformations of solution and gas phase within the crystal structure.^[4]^ Functionally active crystals that possess such modulated phases exhibit charge, bond or chiral ordering, variation in occupancies or destruction and reformation of intermolecular interactions including toggle between chemically different species (like ordering of salt and neutral cocrystal).^[5]^ Thus, the crystal‐chemical analysis of such aperiodic but long‐range ordered compounds provides not only the understanding of variations of intermolecular interactions within unique temperature and/or pressure points, but also presents information on synergistic effects of different types of these interactions on crystal packing and symmetry. Such “exotic” interplay of chemical bonds can be exploited to design potential functional molecular materials for various target properties.

**Figure 1:**
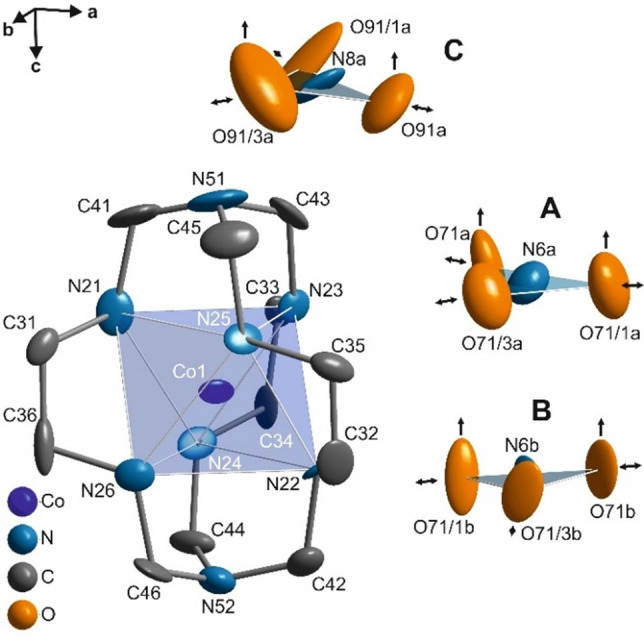
One formula unit of Co(sep)(NO_3_)_3_ with atomic labels emphasizing CoN_6_ octahedra and planar nitrate groups. The vertical arrows and horizontal both sided arrows represent stretching/breathing and out of plane bending modes in Raman spectra reported by Dubicki et al. (1984).^[2a]^ Hydrogen atoms have been omitted for clarity.

Over decades, encapsulated metal ion complexes have demonstrated diverse applications as catalytic agents, in electrochemistry as reductants, photochemistry, water splitting agents, biomedical imaging and pharmaceutics.^[6 and references therein]^ The metal‐organic salt of Λ‐cobalt (III) sepulchrate trinitrate [Co(sep)(NO_3_)_3_ hereon] is one of the earliest synthesized macrobicyclic nitrogen cages for metal ions among (*S*)‐[(1,3,6,8,10,13,16,19‐octaazabicyclo[6.6.6]eicosane) cobalt(III)]^3+^ complexes (Figure 1).^[7]^

**Figure 2:**
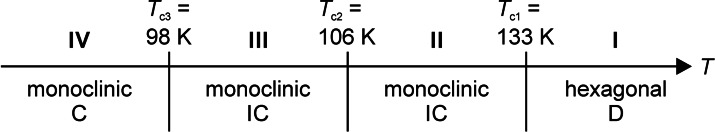
Schematic representation of phase transitions in Co(sep)(NO_3_)_3_ from hexagonal disordered (D) at room temperature via incommensurate (IC) towards a commensurate phase (C) upon cooling.

Co(sep)(NO_3_)_3_ crystallizes in hexagonal space group *P*6_3_22 with lattice parameters, *a*=8.4945 (5) Å, *c*=15.9195 (13) Å, *V*=994.80 (12) Å^3^ at ambient conditions (phase I).^[8]^ The crystal packing is defined by layers of cobalt (III) sepulchrate [Co(sep) hereon] cages and two ordered nitrate groups (nitrate groups A and B) interacting via N−H⋅⋅⋅O bonds. These layers are separated by layers of sixfold orientational disordered nitrate groups (nitrate group C).^[8]^ The crystals exhibit strong dependence of circular dichroism as function of temperature, which is attributed to the convergence of outer sphere nitrate groups A and B towards the Co(sep) cage.^[8a]^ Temperature (*T*) dependent light microscopy revealed a phase transition at *T*_c1_=133 K owing to formations of biaxial domains (phase II).^[9a]^ Temperature‐dependent Raman spectroscopy demonstrated a twofold degeneracy in Co−N coordination and inequivalent frequency modes for nitrate groups A and B below *T*_c1_, suggesting a lowering of the crystal symmetry.^[9a]^ In addition, the phase transition is also associated with freezing of the dynamically disordered nitrate groups C.^[9a]^ *T*‐dependent neutron diffraction confirmed the phase transition with appearance of additional satellite reflections in the diffraction pattern below *T*_c1_.^[9b]^ The positions of the satellite reflections with respect to the main reflections were reported to vary as function of *T*, confirming the incommensurate nature of the modulation wave vector, **q**. Depending on the magnitude of **q**, two other phase transitions have been found at *T*_c2_=106 K (phase III) and *T*_c3_=98 K (phase IV), respectively (Figure 2).^[9b]^ These observations corroborate our single‐crystal X‐ray diffraction (SCXRD) experiments at lower temperatures using synchrotron radiation. Our earlier report confirmed that phase IV is a lock‐in commensurate (C) phase and can be interpreted as an ordered 12‐fold superstructure^[9c]^ (*Z*′=12, where *Z*′=number of independent entities in the asymmetric unit^[10]^) of the room temperature structure (*Z*′=1/6). The formation of the complex superstructure was argued to lie in the avoidance of perturbative C−H⋅⋅⋅O bonds between the Co(sep) cages and nitrate groups A and B, while the ordering of the nitrate groups C is determined by densification of the Co(sep) cages around its co‐ordination sphere.^[9c]^

In the present study we have investigated phase III above the lock‐in phase transition *T*_c3_ at *T*=100 K by means of SCXRD. The IC structure is described within the higher dimensional superspace approach.^[11]^ Molecular distortions and variations are analyzed in comparison to earlier spectroscopic work. We also present the phase diagram consisting of scheme of the intermolecular interactions, synergy and competitions amongst N−H⋅⋅⋅O and C−H⋅⋅⋅O hydrogen bonds and other non‐bonded interactions within the unique parameter defined by phase of the modulation, *t* (Figure 2).

## Results and Discussion

### Symmetry and modulated structure

**Figure 3:**
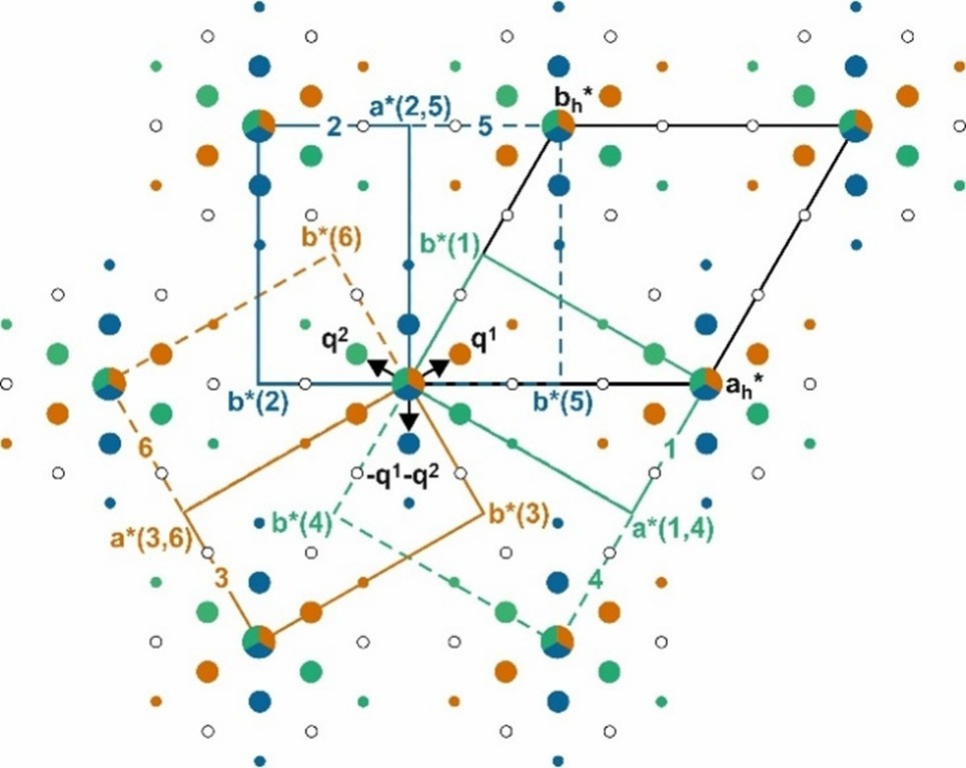
Schematic representation of the six monoclinic pseudo‐orthohexagonal twin domains. Colored filled circles represent the observed reflections while absent ones are shown by empty black circles. **q^1^**, **q^2^** and **q^1^+q^2^** belong to the orange {3,6}, green {1,4} and blue {2,5} domains respectively. Note that the formal relation between the monoclinic pairs (dashed and complete of same color) is twofold rotation parallel **a** axes of the three pseudo‐orthorhombic domains with the c* axes either pointing upwards or downwards perpendicular to the plane of the paper. The assignment of the **q**‐vectors to the twin domains has been chosen consistent with *International Tables for Crystallography, Vols. A and C*.^[13]^

The SCXRD data at *T*=100 K was processed using the software suite *EVAL*15.^[12]^ All reflections (main reflections and satellites reflections up to 2nd order) could be indexed using five integers (*hklmn*) in hexagonal setting with two modulation wave vectors, **q^1^** =(*σ*_h_, *σ*_h_, 0), **q^2^** =(−2*σ*_h_, *σ*_h_, 0) as well as their linear combination **q^3^** =−**q^1^**−**q^2^** =(*σ*_h_, −2*σ*_h_,0) (*σ*_h_ ∼0.088). Reflection statistics revealed that the average intensities of satellites unique to **q^1^**, **q^2^** and **q^3^** are unequal (${\left\langle I\right\rangle }_{q1}:{\left\langle I\right\rangle }_{q2}:{\left\langle I\right\rangle }_{q3}$ ≈10 : 14 : 15). Such difference in intensities contradict a hexagonal symmetry where those satellite reflections are symmetrically equivalent by the sixfold rotation and should be equal in intensity. Furthermore, satellite reflections of mixed 2nd order which belong to combinations of (+**q^1^**, −**q^2^**), (+**q^1^**, −**q^3^**), (+**q^2^**, −**q^3^**), (−**q^1^**, **q^2^**), (−**q^1^**, **q^3^**) and (−**q^2^**, **q^3^**) with indices $\left(hkl1\bar{1}\right)$ , $\left(hkl21\right)$ , $\left(hkl12\right)$ , $\left(hkl\bar{1}1\right)$ , $\left(hkl\bar{21}\right)$ and $\left(hkl\bar{12}\right)$ respectively could not be observed in the diffraction pattern (Figure 3). The observations on inequivalent intensities and missing mixed order satellite reflections bears evidence to the formation of domain structures with lower symmetry as observed in light microscopy and Raman scattering experiments. Furthermore, Bragg peaks of both main and satellite reflections appear to be split.^[9d]^ Therefore, the Bragg peaks were alternatively indexed using three *C*‐centered orthohexagonal cells that are rotated by 120° to each other. Under this transformation, **q^1^**, **q^2^** and **q^3^** belong exclusively to only one of the three different orthorhombic domains (domains 3, 1 and 2 in Figure 3)^[13]^ with *a*_o_=*a*_h_, *b*_o_=√3*b*_h_, *c*_o_=*c*_h_ and **q^o^** =(*σ*_o_,0,0), where *σ*_o_=2*σ*_h_ reducing the description from (3+2)D to (3+1)D modulated structure. Severe peak overlap prevented integration of intensities of the diffraction peaks for individual twin domains (pseudo‐merohedral in nature).

Following a strategy discussed earlier,^[9c]^ data integration was done in the pseudohexagonal cell only for the reflection indices $\left(hkl00\right)$ , (hkl±m0) , (hkl0±n) and (hkl±m∓n) ; m=n≤2 . This dataset was split into four subsets for main (common for all domains) and satellite reflections belonging to domains 1, 2 and 3 respectively. The datasets were averaged in the orthorhombic superspace group *C*222_1_(*σ*_o_00)*s*00.^[14]^ However, the internal agreement factors between intensities of satellite reflections, *R*_int_ are found to be significantly large while their average significance, *R*_σ_ $\left(\left\langle \sigma \left(I\right)\right\rangle /\left\langle I\right\rangle \right)$ are lower (Table 1). In addition, the orthorhombic symmetry requires the nitrate groups A and B (Figure 1) to be related by either the twofold rotation parallel **a** or **b** which contradicts the observations from Raman spectroscopy. Further distortion towards monoclinic symmetry have three possibilities for space groups; (**a**‐unique) *C*2, (**b**‐unique) *C*2 and (**c**‐unique) *C*2_1_ respectively. While the former two space group symmetries render symmetry equivalence of the nitrate groups A and B, they are unrestricted in the later. The four datasets were then averaged in superspace group *C*2_1_(*σ*_1_ *σ*_2_0)0,^[14]^ *σ*_1_≈*σ*_o_, *σ*_2_=0 with three additional twin domains described by twofold rotation parallel to **a** axes of each of the three orthohexagonal domains (Figure 3). The *R*_int_‐values for the satellite reflections are sufficiently lower and agree well with their corresponding *R*_σ_‐values. Re‐determination of the lattice parameters applying indexation with the six *C*‐centered monoclinic twin lattices also revealed small distortion in the monoclinic angle, *γ* [=90°–0.0042(10), Table 3].

**Table 1:** Statistical parameters for observed reflections [*I* >3σ(*I*)] at *T*=100 K averaged in superspace groups (SSG) orthorhombic *C*222_1_(σ00)*s*00 or monoclinic (**c**‐unique) *C*2_1_(σ_1_σ_2_0)0 respectively.

	orthorhombic	monoclinic
*R*_int, all_	0.0475	0.0371
*R*_int, m=0/1/2_	0.0233/0.1313/0.2489	0.0235/0.0970/0.1629
*R*_σ, all_	0.0490	0.0541
*R*_σ, m=0/1/2_	0.0438/0.0824/0.1792	0.0439/0.0916/0.1979

**Table 2:** Comparison of angles (°) within CoN_6_ octahedron in phase I, III and IV.

	Phase I^[a],[b]^	Phase III	Phase IV^[c]^
N21‐Co−N26	87.1	86.0–90.5	86.4–87.0
N22‐Co−N25	87.1	84.5–87.5	85.6–87.1
N23‐Co−N24	87.1	85.4–87.1	87.2–88.0
N22‐Co−N23	90.9	89.7–91.1	90.1–90.3
N21‐Co−N24	90.9	90.2–90.9	90.4–90.8
N25‐Co−N26	90.9	90.2–91.3	90.4–90.7
N22‐Co−N26	91.1	91.0–92.7	90.1–90.9
N23‐Co−N25	91.1	90.5–92.0	90.6–90.8
N24‐Co−N26	91.1	91.6–92.6	90.8–91.5
N22‐Co−N24	91.1	90.4–93.1	90.4–91.3
N21‐Co−N25	91.1	90.2–92.2	91.8–92.7
N21‐Co−N23	91.1	88.2–92.3	92.3–92.9

[a] Ref. [1b]; [b] N21 through to N26 are symmetry equivalent by 3_2_ || **c**, [c] Ref. [2c].

**Table 3:** Experimental and crystallographic data of phase III.

Crystal data	
chemical formula	[Co(C_12_H_30_N_8_)]_3_^+^ ⋅ 3 NO_3_^−^
*M*_r_	531.39
crystal dimensions [mm]	0.30×0.15×0.10
crystal system	monoclinic (**c**‐unique)
Superspace group	*C*2_1_(*σ*_1_ *σ*_2_0)0
*T* [K]	100
*a* [Å]	8.4181(3)
*b* [Å]	14.5950(2)
*c* [Å]	15.6886(2)
γ [°]	89.9958(10)
*V* [Å^3^]	1927.54(8)
*Z*	4
wavevector	**q**=0.176679(21)a*
radiation type	synchrotron
Wavelength [Å]	0.60
*μ* [mm^−1^]	0.609
Diffraction data	
[sin(*θ*)/*λ*]_max_ [Å^−1^]	0.77
▵*ϕ* [°]	1
exposure time [s]	10, 20, 30, 40, 60, 120
absorption correction	empirical, multiscan
criterion on observability	*I*>3*σ*(*I*)
Unique reflections	
all (obs/all)	20748/64524
*m*=0	4351/4919
|*m*|=1	14101/29687
|*m*|=2	2300/29918
*R*_int_ (obs/all)	0.0371/0.0446
Refinement	
*GoF* (obs/all)	1.90/1.19
*R*_obs_/*wR*_all_	
All	0.0680/0.0874
*m*=0	0.0495/0.0633
|*m*|=1	0.0808/0.0887
|*m*|=2	0.1204/0.1855
No. of parameters	660
Twin volumes (1,2,3,4) (5,6)	0.1873(19), 0.2130(9), 0.1092(8), 0.1787(9), 0.1887(9), 0.1230(8)
▵*ρ*_min_/▵*ρ*_max_ [eÅ^−3^]	−1.84/1.57

Structure refinement of the (3+1)D modulated structures was performed using the software suite *JANA*2006^[15]^ applying distance restraints within the Co(sep) cage. Angles between the six Co−N_lig_ co‐ordination bonds were unrestrained to compare the distortions of the CoN_6_ octahedron to that in phase I and IV respectively, while the covalent bond geometries between the ligand nitrogen (N_lig_), ethylene (C_eth_) and apical carbons (C_ap_) and capping nitrogen atoms (N_ap_) were restrained (N_lig_=N21–N26, C_eth_=C31–C36, C_ap_=C41–C46, N_ap_=N51, N52). Nitrate groups A and B were described using a rigid body model at two different sites with ideal trigonal planar geometry in point symmetry 32, while angle and distance restraints were applied for nitrate group C. Positions and anisotropic atomic displacement parameters (ADP) for the atoms of Co(sep) cage and nitrate group C with displacive modulation described up to 2nd order harmonic order were refined while TLS parameters^[16]^ and 2nd harmonic order for translation and rotations for the rigid bodies were refined (Figure S1, further detailed in Supporting Information). The partial *R*‐factors for the satellite reflections [*R*_obs_ (|*m*|=1)=0.0808, *R*_obs_ (|*m*|=2)=0.1204] agree well with their corresponding *R*_int_ and *R*_σ_ values (Table 1, Table 3).

### Phase transitions and phase relations

**Figure 4:**
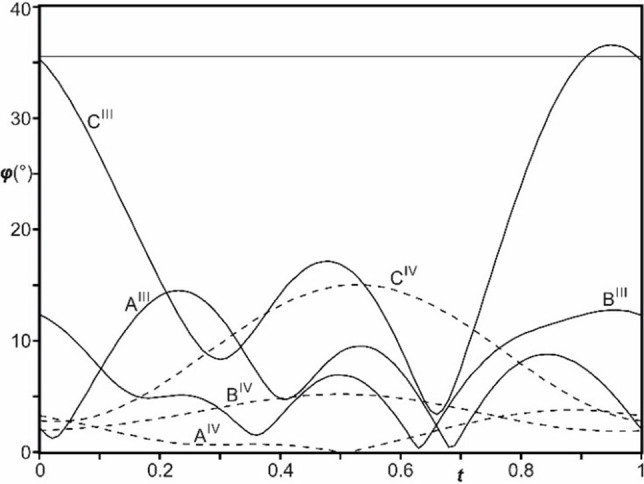
*t*‐Plot of the dihedral angles *ϕ* (°) between the planes of nitrate groups A, B and C and the (**a**,**b**)‐plane in phase III (full curves) and phase IV^[9c]^ (dashed curves). Horizontal line corresponds to the orientation of sixfold disordered nitrate group C planes in phase I. Orientation of the planes of A and B are *ϕ*=0° in phase I.

**Figure 5:**
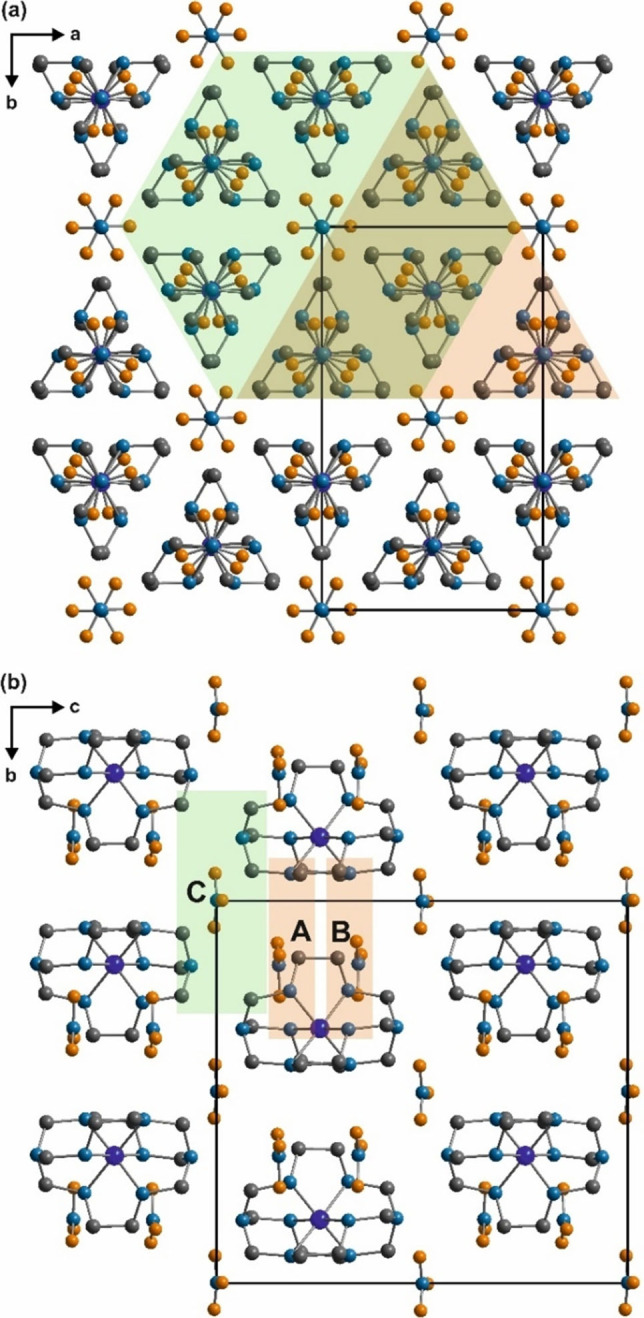
(a) View along (001) and (b) along (100) of the basic structure contents of non‐hydrogen atoms of Co(sep)(NO_3_)_3_ in phase III drawn up to −1≤*x*≤1, −1/2≤*y*≤1 and −1/2≤*z*≤1/2 depicting the co‐ordination environments of nitrate groups A, B and the Co(sep) cages (transparent orange overlay); and nitrate groups C and the Co(sep) cages (transparent green overlay).

Space group symmetries from the disordered phase I down to the lock‐in phase IV follow group‐subgroup relations via lowering of symmetry from hexagonal to monoclinic below *T*_c1_, while the monoclinic symmetry is retained below *T*_c3_ with the lock‐in of **q**. The basic structure of phase III is allover quite similar to the one of phase I albeit the nitrate group C is ordered (Figure 5, Figures S2–S6). Modulation of displacements (*u*) are found to be pronounced with amplitude as large as 0.5 Å and in the order |*u*|_cosep cage_<|*u*|_nitrate B_<|*u*|_nitrate A_<|*u*|_nitrate C_ (Table S2). An important distinction between the lock‐in phase IV and the present crystal structure is the absence of second order harmonics for displacive modulation in the former. This implies that the distortions in phase III are half the period to that of phase IV. Within the cage, distortion of the CoN_6_ co‐ordination from an ideal octahedral geometry are found to be larger as compared to phase I (Table 2). The rather pronounced and unequal variations within individual N_lig_−Co−‐N_lig_ angles (▵_min_/▵_max_=0.7°/4.5°) is in agreement with the splitting of *T*_2*g*_ normal modes of [CoN_6_] in Raman spectra below *T*_c1_.^[2a]^ Nitrate group C exhibits large modulations in the orientation of its plane with respect to (001) plane. The variations are also large (dihedral angle, 3.4°≤*ϕ*_nitrate C_≤36.6° in Figure 4; compare to *ϕ* ^I^ ≈35.6°^[8b]^) while these rotations are suppressed in phase IV (*ϕ* ^IV^≤15°^[9c]^). Rotations out of the (001) plane were also found to be significant for nitrate groups A and B (0.4°≤*ϕ*_nitrate A_≤14.5° and 0.3°≤*ϕ*_nitrate B_≤12.8° in Figure 4). These distortions are remarkably larger than those in phase IV (*ϕ*_nitrate A,B_≤5°). Notably in the present structure, the pronounced differences in the orientations of the nitrate groups A and B with respect to (a*,b*)‐plane as function of *t* is in agreement with the monoclinic distortion (*γ*≠90°) and complements the double degeneracy of the stretching and bending mode frequencies in Raman spectra below *T*_c1_.^[9a]^

**Figure 6:**
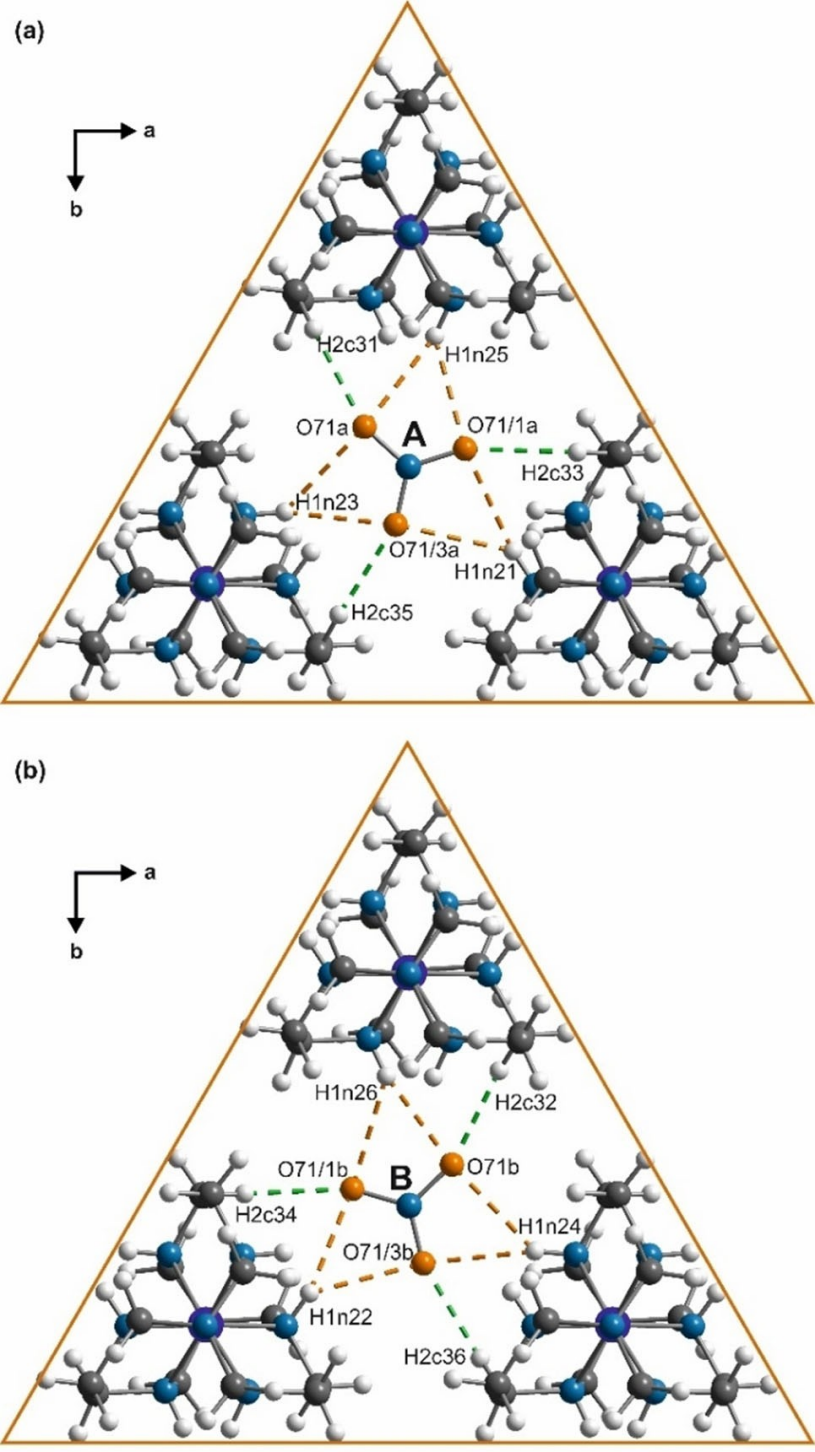
Part of basic structure (compare with Figure 5) showing scheme of intermolecular N−H⋅⋅⋅O bonds (dashed orange) and C−H⋅⋅⋅O bonds (dashed green) between nitrate groups A, B and Co(sep) cages. Nitrate groups A and B; and Co(sep) cages are centred at *z*=0.15, *z*=0.34 and *z*=0.25 respectively.

**Figure 7:**
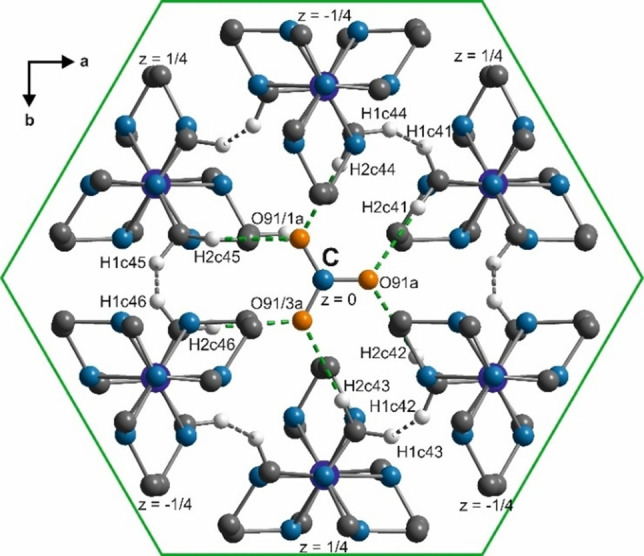
Scheme of C−H⋅⋅⋅O bonds (dashed green) between the six surrounding Co(sep) cages and the nitrate group C; and H⋅⋅⋅H contacts (dashed grey) between the Co(sep) cages (compare with Figure 5). Only relevant hydrogen atoms are drawn.

Also, variation in non‐bonded distances between the Co atom and the lighter atoms within the cage; and between Co atoms and centers of nitrate groups A, B and C are larger as compared to phase IV (Figures S2 to S6). Larger distortions within the CoN_6_ octahedra and the significant deviations of the molecular planes of the nitrate groups A and B is, on first impression, evidence of pronounced frustrations in the conformations of the intermolecular hydrogen bonds as compared to IV (Figure 5, Figure 6, Figure 7). The unique property of the nitrate group C that it retains the conformation of phase I at a certain unique *t* value hints at the intermediary nature of the IC phase III. Investigations into the competitive intermolecular interactions in the present structure are done by mapping them onto those of phase IV.

### Enhanced frustrations of intermolecular interactions in incommensurate phase III versus lock‐in phase IV

Nitrate groups A and B are each coordinated by three Co(sep) cages. The oxygen atoms of A and B anions are involved in bifurcated N−H⋅⋅⋅O hydrogen bonds (*d*_H⋅⋅⋅O_≈2.5 Å in phase I^[8b]^) with the amine N_lig_H groups (Figure 6).

**Figure 8:**
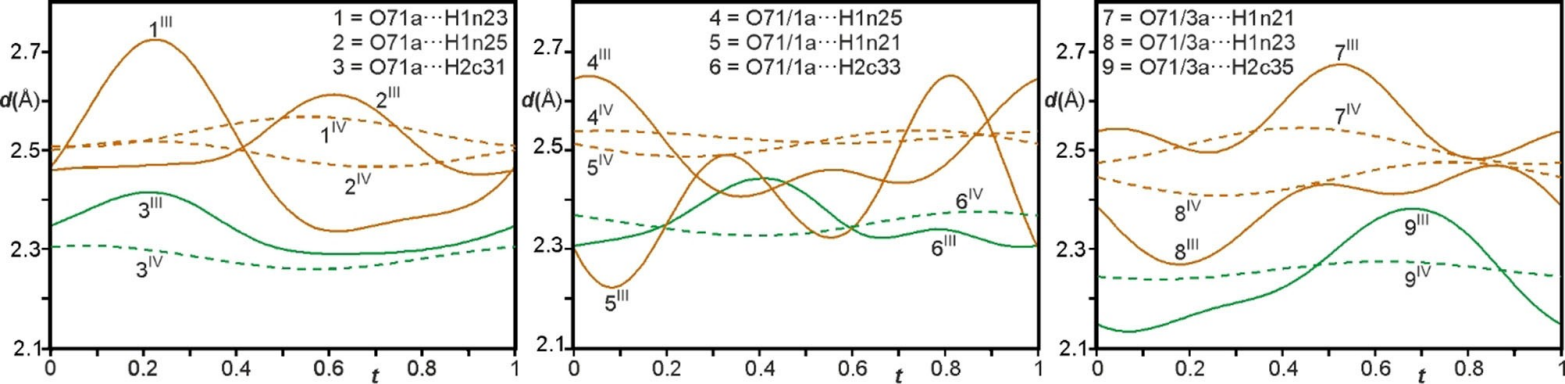
t‐Plots of the interatomic distances (Å) between the oxygen atoms of nitrate group A and hydrogen atoms of the Co(sep) cage involved in N−H⋅⋅⋅O bonds (orange) and C−H⋅⋅⋅O bonds (green) in phase III (full curves) compared to those in phase IV^[9c]^ (dashed curves). A similar plot for nitrate group B is given in Supporting Information, Figure S7.

The variations in these H⋅⋅⋅O distances are large (∼2.2–2.7 Å, Figure 8, Figure S7, Table S3) and more than twice that for the 12‐fold superstructure (2.4–2.6 Å^[9c]^).

Within each of the bifurcated bond pairs with a unique oxygen atom of the nitrates, the change in distances are complimentary to each other. While these interactions are long and weak for typical highly directional N−H⋅⋅⋅O bonds,[^17a^, ^17b^] the bifurcated geometry possibly accounts for such long H⋅⋅⋅O distances.[^17c^, ^17d^] In addition to the N−H⋅⋅⋅O bonds, nitrate groups A and B and the Co(sep) cages are also involved in weak C−H⋅⋅⋅O bonds where the C_eth_−H groups act as donors of hydrogens (Figure 6).

**Figure 9:**
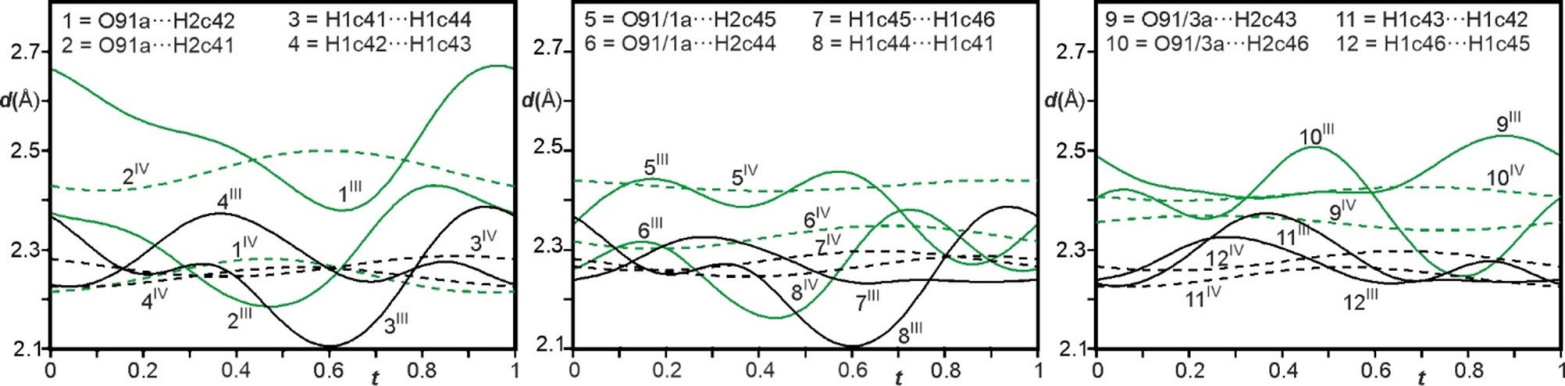
*t*‐Plots of the interatomic distances (Å) between the oxygen atoms of nitrate group C and Co(sep) cage involved in C−H⋅⋅⋅O bonds (green); and between hydrogen atoms of adjacent Co(sep) cages (black) in phase III (full curves) compared to those in phase IV^[9c]^ (dashed curves).

In spite of the weakly polarizable nature of these CH groups (sp^3^ hybridized), the C−H⋅⋅⋅O bonds are short amongst those reported.^[18]^ The variations in the distances (∼2.1–2.4 Å, Figure 9, Figure S7) are significantly lower compared to the N−H⋅⋅⋅O bonds but larger than those in phase IV^[9c]^. Most importantly, unlike the concomitant behavior (as function of *t*) of the weak and strong hydrogen bonds in phase IV, here the H⋅⋅⋅O distances involving C−H⋅⋅⋅O bonds are equal and even larger than those involved in N−H⋅⋅⋅O bonds at certain *t*‐sections which shows that the former can be stronger when the later are weaker and vice‐versa. This observable competition between the N−H⋅⋅⋅O and C−H⋅⋅⋅O bonds accounts for such large rotations of the planes of these nitrate groups A and B out of the (**a,b**)‐plane in the present structure.

The ordering of the nitrate group C in phase III is associated with three times as large rotations out of the (**a,b**)‐plane compared to those of A and B (Figure 4). While evidence for C−H⋅⋅⋅O bonds between nitrate group C and the six surrounding Co(sep) cages was found in phase IV, here the variations are significantly larger (*d*_H⋅⋅⋅O_ ∼2.2–2.7 Å compare ∼2.2–2.5 in phase IV, Table S3). The reformation of these attractive C−H⋅⋅⋅O bonds in phase III possibly governs ordering of these nitrate anions. However, these hydrogen bonds require densification of the Co(sep) cages which results in alarmingly short H⋅⋅⋅H contacts between adjacent Co(sep) cages (*d*_H⋅⋅⋅H_ ∼2.1–2.4 Å in Figure 7, Figure 9). Apparently, the tendency of the plane of the nitrate group C to align itself with the (001) plane complements these short H⋅⋅⋅H contacts. This indicates that steric factors competing with the C−H⋅⋅⋅O bonds results in strong modulation of the nitrate group C in phase III.^[19]^ Although the rotation of this nitrate group C is largely suppressed in phase IV, the modulation still remains to be largest at the site of this anion.^[9c]^

A unique property of phase III is that the C−H⋅⋅⋅O bonds at different sites appear to be uncorrelated because the variations of those involving nitrate group A and B and that of C are different. In the lock‐in phase, these changes are nearly equal. In addition, the tendency of the N−H⋅⋅⋅O bonds to become shorter than the C−H⋅⋅⋅O bonds depict that the stability of the intermediary phase III is also governed by the N−H⋅⋅⋅O bonds in addition to the C−H⋅⋅⋅O bonds, while the later plays the dominant role in phase IV.^[9c]^

## Conclusion

The incommensurately modulated structure of Co(sep)(NO_3_)_3_ in the intermediary phase III has been successfully described within (3+1)D superspace. The significant distortions in the molecular conformations of the Co(sep) cage and the nitrate groups corroborate observations from Raman spectroscopy. Like many charge density wave order compounds,^[20]^ the relations in the different phase sequences (phase I to III towards IV) could be uniquely established by convolution of atomic modulation functions on the ordered model of disordered phase I. The presence of larger amplitude of modulation with contributions from additional second order harmonics for displacive modulations in phase III compared to lock‐in phase IV is due to significant competitions between intermolecular N−H⋅⋅⋅O and C−H⋅⋅⋅O hydrogen bonds; and between intermolecular C−H⋅⋅⋅O bonds and short H⋅⋅⋅H interactions.

The freezing of the disorder below *T*_c1_ into inequivalent sites is understood in terms of order in the conformation of the nitrate group C within the monoclinic symmetry of the six twin domains with unequal volumes. The large modulations of these nitrates along *t* fulfilling both the rotations out of (**a**,**b**) plane in phase I and phase IV depicts the intermediary character of phase III. The origin and stability of incommensurability in phase III preceding the lock‐in phase can be explained with respect to gain in orientational order of the disordered anions at the cost of severe competitions between different intermolecular interactions. These frustrations in the crystal packing are sufficiently suppressed in the lock‐in phase aiding in the formation of the ordered *Z*′=12 twelvefold superstructure of the disordered structure in phase I.

## Experimental Section

Single crystals of Co(sep)(NO_3_)_3_ were synthesized in the research group of Alan M. Sargeson;^[22]^ the crystals are at ambient conditions stable in air.

Temperature of the crystal was maintained by an open flow nitrogen‐gas cryostat by Oxford Cryosystems. Single‐crystal X‐ray diffraction experiments were performed at *T*=100 K using a four‐circle Euler geometry diffractometer at beamline D3 in Hasylab DESY, Hamburg, Germany. Data collection was done by *ϕ* scans at detector offsets 2*θ*=0° and 30°, with exposure times 10 s, 20 s, 30 s, 40 s, 60 s and 120 s employing a radiation of wavelength, *λ*=0.60 Å using MAR‐CCD detector. Determination of lattice parameters and data integration was done using *EVAL*15.^[12]^ Absorption correction was performed using *SADABS*,^[21]^ in point group symmetry 2/*m*. Structure refinements were performed using the software suite *JANA*2006.^[15]^

Deposition Number jBag759KAES contains the supplementary crystallographic data for this paper. These data are provided free of charge by the B‐IncStrDB at the Bilbao Crystallographic Server.

## Conflict of interest

The authors declare no conflict of interest.

1

## Supporting information

As a service to our authors and readers, this journal provides supporting information supplied by the authors. Such materials are peer reviewed and may be re‐organized for online delivery, but are not copy‐edited or typeset. Technical support issues arising from supporting information (other than missing files) should be addressed to the authors.

Supporting Information

## Data Availability

The data that support the findings of this study are available in the supplementary material of this article.
