# Supplementary material for: Incommensurate Phase in Λ‐cobalt (III) Sepulchrate Trinitrate Governed by Highly Competitive N−H⋅⋅⋅O and C−H⋅⋅⋅O Hydrogen Bond Networks
Source: Chemistry. 2022 Feb 10;28(13):e202104151. doi: 10.1002/chem.202104151 (PMC9303887; doi:10.1002/chem.202104151)
Supplement: Supplementary file 1 — Supporting Information [file CHEM-28-0-s001.pdf]

Chemistry–A European Journal

Supporting Information

Incommensurate Phase in Λ-cobalt (III) Sepulchrate Trinitrate Governed by Highly Competitive N–H···O and C–H···O Hydrogen Bond Networks

Somnath Dey,* Andreas Schönleber,* Sander van Smaalen, Wolfgang Morgenroth, and Finn Krebs Larsen

Author Contributions

S.D. Data curation:Lead; Investigation:Equal; Software:Equal; Validation:Equal; Writing – original draft:Lead
A.S. Conceptualization:Equal; Data curation:Equal; Funding acquisition:Equal; Investigation:Equal; Project administration:Equal; Software:Equal; Supervision:Equal; Validation:Equal; Writing – original draft:Equal
S.v. Conceptualization:Equal; Funding acquisition:Equal; Investigation:Equal; Project administration:Equal; Supervision:Equal; Validation:Equal; Writing – original draft:Equal
W.M. Investigation:Supporting; Validation:Supporting; Writing – original draft:Supporting
F.L. Investigation:Supporting; Validation:Supporting; Writing – original draft:Supporting

Table of Contents

Section S1. Structure refinement of the incommensurately modulated structure at T = 100 K in phase III.

Figure S1. Higher-dimensional Fourier maps centered at the cobalt atom.
Figure S2. *t*-Plot of interatomic distances between cobalt and ligand nitrogen atoms.
Figure S3. *t*-Plot of interatomic distances between cobalt and ethylene and apical carbon atoms.
Figure S4. *t*-Plot of interatomic distances between cobalt and apical nitrogen atoms.
Figure S5. *t*-Plot of interatomic distances between cobalt and centers of nitrate groups A and B.
Figure S6. *t*-Plot of interatomic distances between cobalt and center of nitrate groups C.
Figure S7. *t*-Plot of interatomic distances between hydrogen of Co(sep) cage and oxygen atoms of nitrate group B involved in hydrogen bonds.

Table S1. Statistical parameters for refinement for different structural models in phase III.
Table S2. Amplitude of atomic modulation functions of non-hydrogen atoms.
Table S3. Intermolecular N–H···O and C–H···O hydrogen bond motifs.

Section S1. Structure refinement of the incommensurately modulated structure at T = 100 K in phase III.

Structure refinements have been performed using the software suite *JANA*2006.[1]

Basic co-ordinates of all atoms were obtained from the model at T = 95 K (phase IV[2]). Within this model ('a' in Table S1), distances and angles between atoms within the cages have been restrained to values those reported at ambient conditions.[3] Nitrate A and B have been described as a single rigid body model at two different positions while a second rigid body model has been described for nitrate group C. Internal geometry of these rigid entities have been defined to be trigonal planar with the nitrogen atoms at the center and oxygen atoms at the apices in point group 321. Positions and isotropic displacement parameters (ADPs) of the atoms were then refined against main reflections at T = 100 K [R_{obs} (m = 0) = 0.1409]. In the next step, first order harmonic waves for displacive modulation of the atoms inside the cage; translation and rotational modulation for the rigid bodies were introduced. Refinement against main and satellite reflections of first order improved the statistical parameters for the main reflections significantly [R_{obs} (m = 0) = 0.0789, R_{obs} ($|m|$ = 1) = 0.1036]. The residual values for main and satellite reflections of first order further improved upon introduction of second order harmonic waves for displacive modulation for atoms; translation and rotational modulation for the rigid bodies [R_{obs} (m = 0) = 0.0785, R_{obs} ($|m|$ = 1) = 0.0964, R_{obs} ($|m|$ = 2) = 0.1384, $\Delta\rho_{min}/\Delta\rho_{max}$ (e/Å^3) = -2.18/2.80]. Refinement of anisotropic ADPs for the atoms and TLS parameters[4] for the nitrate groups improved the residual values significantly [R_{obs} (m = 0) = 0.0533, R_{obs} ($|m|$ = 1) = 0.0826, R_{obs} ($|m|$ = 2) = 0.1300, $\Delta\rho_{min}/\Delta\rho_{max}$ (e/Å^3) = -2.25/1.76]. However, ADPs of three non-hydrogen atoms were found to be non-positive definite. Subsequent refinement of the structural model introducing first order harmonic for modulation of anisotropic ADPs and 3rd order anharmonic ADPs[3] for the cobalt atom improved the fit to the diffraction data [**model 'a'**: R_{obs} (m = 0) = 0.0504, R_{obs} ($|m|$ = 1) = 0.0818, R_{obs} ($|m|$ = 2) = 0.1241, Table S1].

However, ADPs of one non-hydrogen atom was still found to be non-positive definite. In addition, analysis of bond distance and angles revealed that trigonal planar symmetry is not satisfied for the nitrate group C (unequal N–O distances and ∠O–N–O ≠ 120 deg for all t-sections). A possible reason for such geometric variations is that the nitrate group C is strongly modulated (Table S2) and rectilinear approximation of rigid body approach in *JANA*2006 cannot guarantee rigid conditions. Within the rigid body approach, additional restraints on angles and distances to maintain trigonal planar geometry led to worse fit to the diffraction data [**model 'b'**: R_{obs} (m = 0) = 0.0523, R_{obs} ($|m|$ = 1) = 0.0859, R_{obs} ($|m|$ = 2) = 0.1323, Table S1].

In the next step, nitrate group C was described as independent atom model with distance and angle restraints to maintain the trigonal planar geometry. Refinement led to better statistical parameters for refinement [**model 'c'**: R_{obs} (m = 0) = 0.0500, R_{obs} ($|m|$ = 1) = 0.0808, R_{obs} ($|m|$ = 2) = 0.1205, Table S1] and physically meaningful model.

In the final step, the parameter for isotropic extinction correction was refined that led to improved residual values for the main reflections [R_{obs} (m = 0) = 0.0495, R_{obs} ($|m|$ = 1) = 0.0808, R_{obs} ($|m|$ = 2) = 0.1204]. The outstanding density in the final structural model is due to residual features near the cobalt atom (Fig S1).

Attempts to refine second order Fourier waves for anisotropic and 3rd order anharmonic ADPs including basic parameters for the fourth order anharmonic terms[5] led to large correlations among structural parameters and non-positive definite ADPs for the Co atom. Structure refinement by replacing with the riding model constraints on the hydrogen atoms of N_{lig} atoms by restraints [$d_{N–H}$ = 0.87 ± 0.02 Å and $\angle_{(H–N–Co)} = \angle_{(H–N–C3)} = \angle_{(H–N–C4)}$ = 109.47 ± 1°] resulted in no improvement of the residual values and non-positive definite ADPs for one non-hydrogen atom and was thus discarded from further considerations.

Due to the strong displacive modulation the local environment of the individual atoms is strongly changing with respect to interatomic (intermolecular) distances. Therefor the ADPs are supposed to be modulated, too. Nevertheless, due to the already quite sophisticated model (modulation and twinning) and due to some remaining disorder and less than perfect crystal/diffraction data quality any attempt to introduce ADP modulation for the non-Co atoms was not successful. Consequently, as seen also in Fig. 1, some of the atoms exhibit a large parameter along one direction ("cigar shaped", e.g. O91/1a, N8a, C34) or a short one along one direction ("discus shaped", e.g. N25, N22).

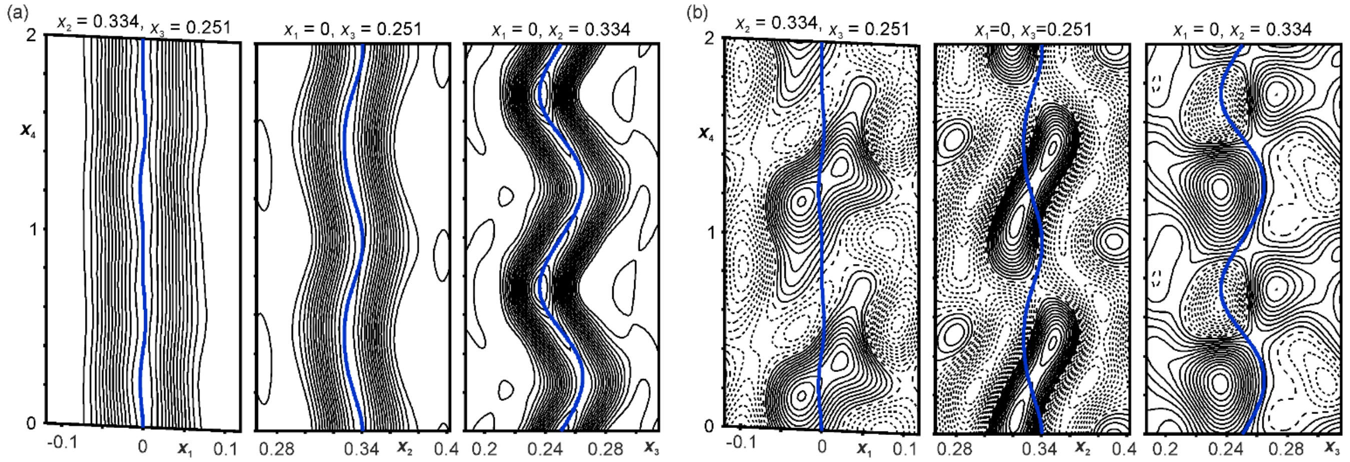

Figure S1. (a) (x_i, x_4) sections (i = 1, 2, 3) of the Fourier map centred at the position of the cobalt atom described with harmonic waves up to 2nd order at T = 100 K. The width of each maps is 2 Å. Contour lines of equal density are at intervals of 1.0 eÅ^{-3}. (b) (x_i, x_4) sections (i = 1, 2, 3) of the difference Fourier map (F_{obs} – F_{calc}) centred at the position of the cobalt atom. Harmonic waves of first order for both harmonic ADPs and 3rd order anharmonic ADPs have been described for the cobalt atom. Residual density $\Delta\rho_{min}/\Delta\rho_{max}$ (eÅ^3) is -1.84/1.57. The step of the contour lines is 0.1 eÅ^{-3}, the width of the maps is 2 Å.

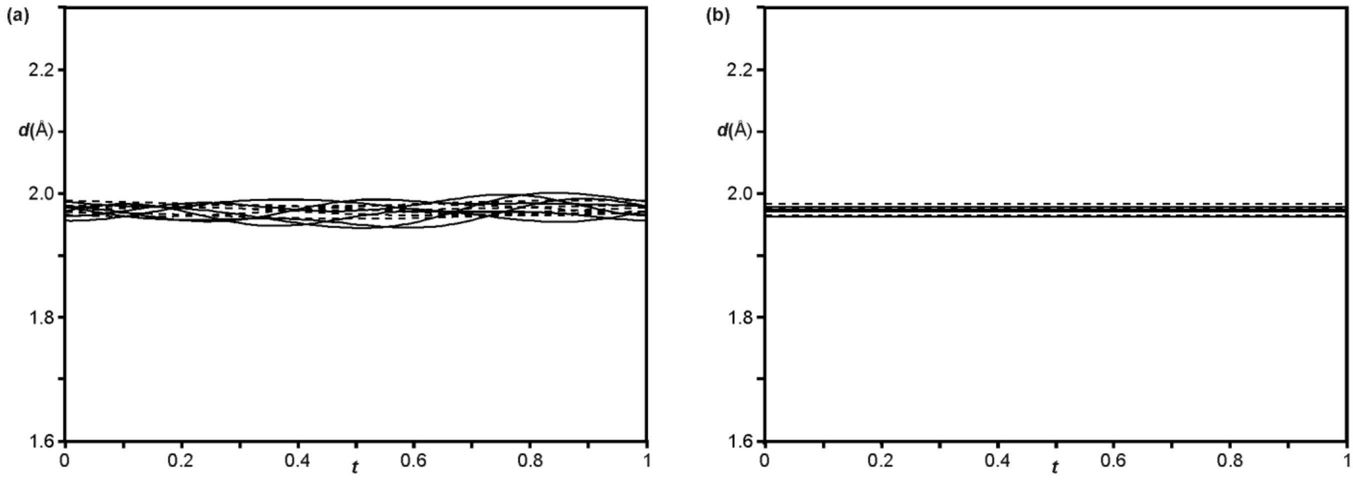

Figure S2. t-Plot of interatomic distances (Å) between Co1 atom and the six co-ordinated N_{lig} atoms inside the Co(sep) cage drawn for the (a) modulated structures versus (b) basic structures respectively; in phase III (full curves and lines) and phase IV[4] (dashed curves and lines).

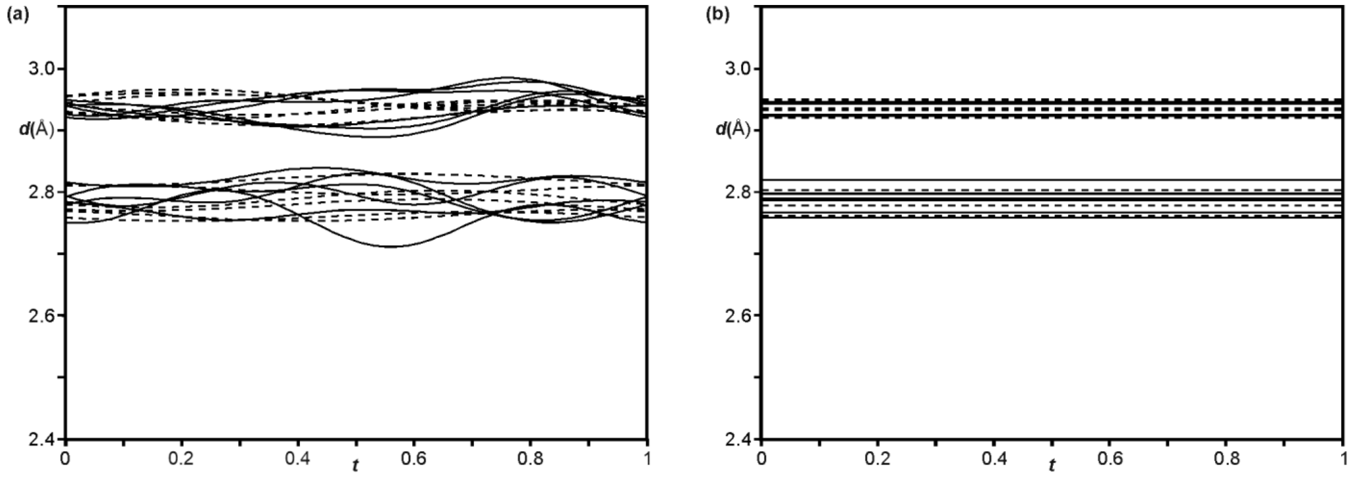

Figure S3. *t*-Plot of non-bonded interatomic distances (Å) between Co1 atom and the six C_{eth} and between Co1 atom and six C_{ap} atoms inside the Co(sep) cage drawn for the (a) modulated structures versus (b) basic structures respectively; in phase III (full curves and lines) and phase IV[4] (dashed curves and lines).

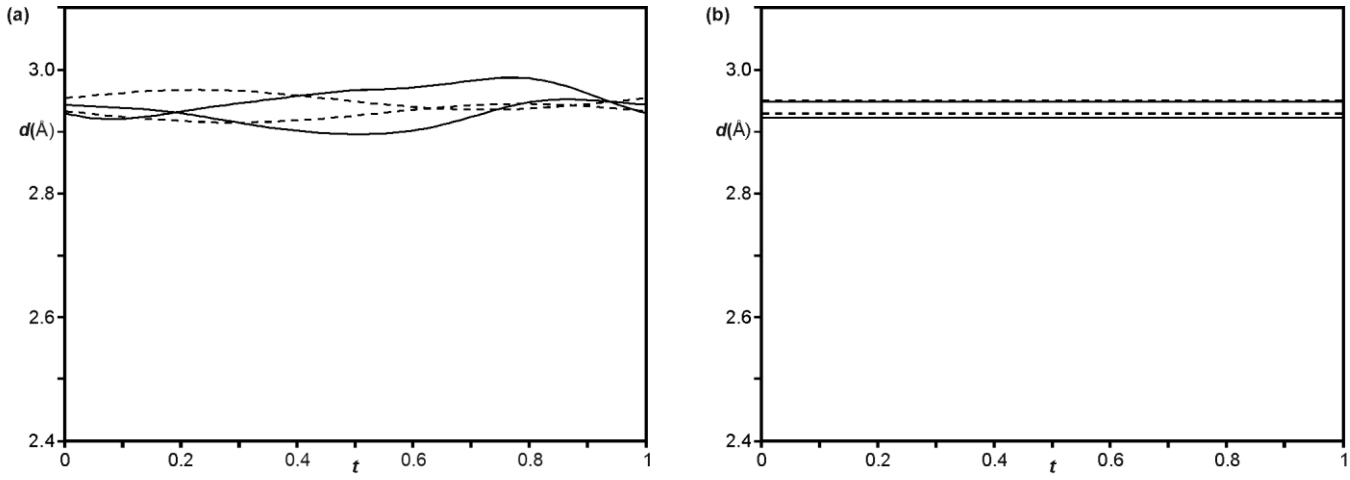

Figure S4. *t*-Plot of non-bonded distances (Å) between Co1 atom and the two apical N5 atoms inside the Co(sep) cage drawn for the (a) modulated structures versus (b) basic structures respectively; in phase III (full curves and lines) and phase IV[4] (dashed curves and lines).

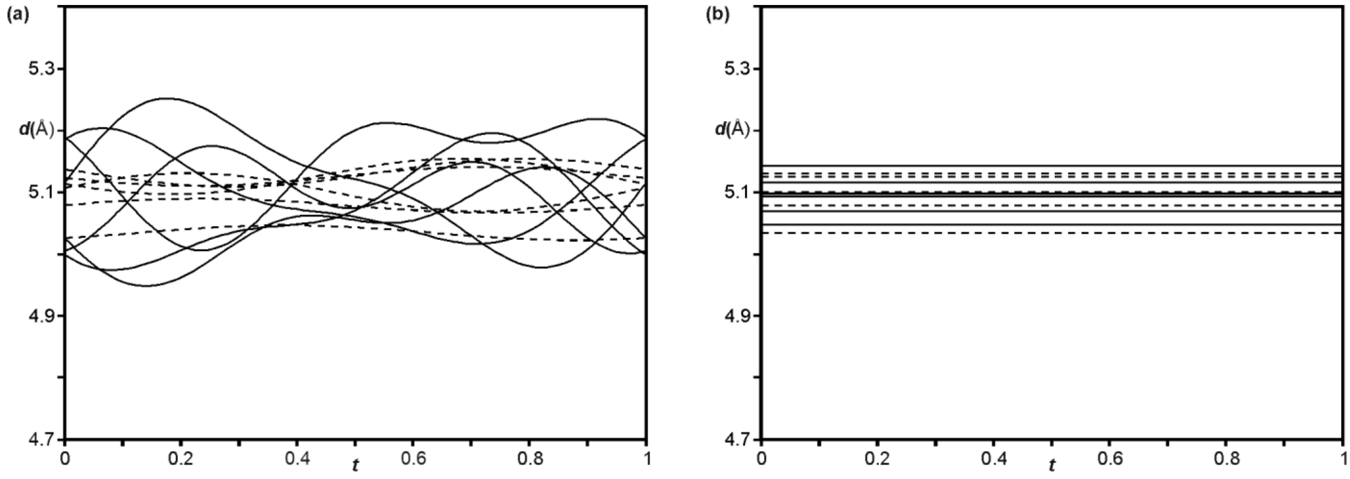

Figure S5. *t*-Plot of non-bonded distances (Å) between Co1 atom and the three each N6 atoms of nitrate groups A and B for the (a) modulated structures versus (b) basic structures respectively; in phase III (full curves and lines) and phase IV[4] (dashed curves and lines).

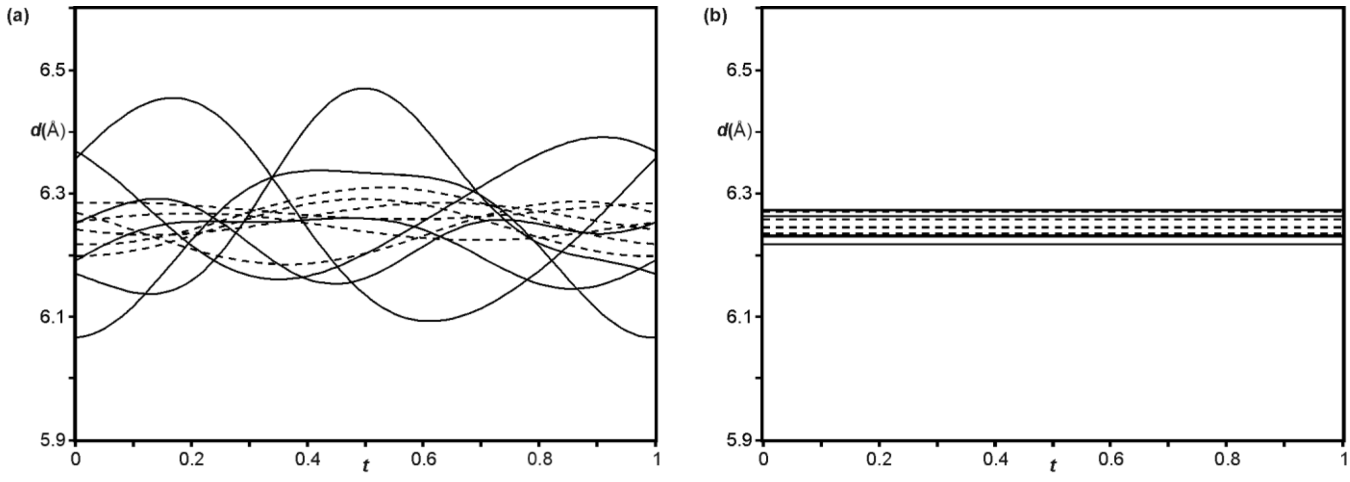

Figure S6. *t*-Plot of non-bonded distances (Å) between Co1 atom and the six N8 atoms of nitrate groups C for the (a) modulated structures versus (b) basic structures respectively; in phase III (full curves and lines) and phase IV[4] (dashed curves and lines).

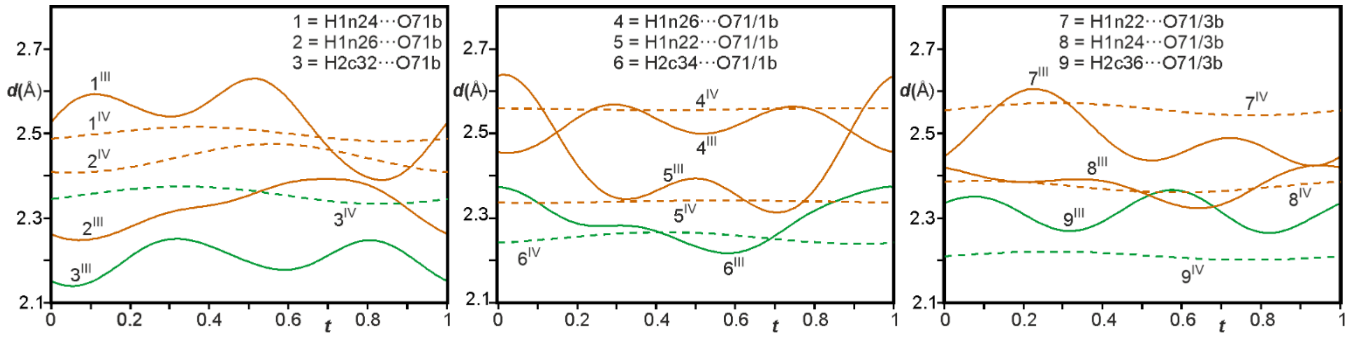

Figure S7. *t*-Plots of the interatomic distances (Å) between the oxygen atoms of nitrate group B and hydrogen atoms of the Co(sep) cage involved in N–H···O bonds (orange) and C–H···O bonds (green) in phase III (full curves) compared to those in phase IV[4] (dashed curves).

Table S1. Comparison of statistical parameters for refinement and validity of the structural models 'a', 'b' and 'c' at T = 100 K. Description of the different models discussed in detail in Section S1: Structure refinement.

	model 'a'	model 'b'	model 'c'
GoF (obs/all)	1.94/1.22	2.07/1.30	1.89/1.19
R_{obs} (all)	0.0691	0.0723	0.0682
R_{obs} (m = 0)	0.0504	0.0523	0.0500
*R*obs |m = 1|	0.0818	0.0859	0.0808
R_{obs} |m = 2|	0.1241	0.1323	0.1205
$\Delta\rho_{min}/\Delta\rho_{max}$ (e/Å^3)	-1.88/1.53	-2.08/1.32	-1.91/1.53
No. of parameters	624	624	659
No. of atoms with npd ADPs	1	2	none

Table S2. Amplitudes of the atomic modulation functions along the three basis vectors in phase III compared to those in phase IV[4]. u^i_1 and u^i_2 (i = a, b, c) represent amplitude for the first (1) and second order harmonic (2) respectively. In phase IV, only u^i_1 is present.[4] Similar to phase IV, the u^i_1 amplitudes are maximum along **c**, largest being 0.5334 Å for atom O91/3a (compare to 0.3091, 0.2966 and 0.3665 Å for atoms C36, O71/1b and O71/3a respectively). For the second order harmonic, the amplitudes (u^i_2) along **c** are not necessarily the largest.

Atom	along **a** (Å)			along **b** (Å)			along **c** (Å)		
	Phase III		Phase IV[4]	Phase III		Phase IV[4]	Phase III		Phase IV[4]
	u^a_1	u^a_2	u^a_1	u^b_1	u^b_2	u^b_1	u^c_1	u^c_2	u^c_1
Co(sep) cage									
Co1	0.0199	0.0130	0.0039	0.0964	0.0087	0.0209	0.2223	0.0138	0.0652
N21	0.0304	0.0324	0.0075	0.1335	0.0382	0.0314	0.2672	0.0576	0.0708
N22	0.0331	0.0318	0.0075	0.1104	0.0182	0.0368	0.2156	0.0311	0.0770
N23	0.0281	0.0466	0.0128	0.0961	0.0348	0.0332	0.1559	0.0517	0.0454
N24	0.0388	0.0385	0.0081	0.1118	0.0195	0.0300	0.1393	0.0507	0.0533
N25	0.0161	0.0335	0.0094	0.0974	0.0326	0.0288	0.2946	0.0328	0.0844
N26	0.0340	0.0379	0.0122	0.1147	0.0204	0.0330	0.2913	0.0412	0.0960
C31	0.0280	0.0201	0.0062	0.1099	0.0290	0.0226	0.3055	0.0695	0.0996
C32	0.0143	0.0289	0.0114	0.1094	0.0078	0.0381	0.2913	0.0585	0.0824
C33	0.0152	0.0284	0.0197	0.1035	0.0250	0.0277	0.0894	0.0730	0.0365
C34	0.0236	0.0204	0.0049	0.1061	0.0186	0.0259	0.0882	0.0717	0.0366
C35	0.0101	0.0206	0.0074	0.0958	0.0145	0.0185	0.2858	0.0647	0.0914
C36	0.0224	0.0244	0.0111	0.1000	0.0203	0.0266	0.3091	0.0618	0.0934
C41	0.0484	0.0735	0.0211	0.1691	0.0535	0.0607	0.2517	0.0510	0.0778
C42	0.0788	0.0656	0.0163	0.1878	0.0475	0.0500	0.2222	0.0306	0.0764
C43	0.0525	0.0764	0.0301	0.1557	0.0653	0.0409	0.1796	0.0360	0.0607
C44	0.0840	0.0623	0.0185	0.1747	0.0541	0.0538	0.1399	0.0467	0.0603
C45	0.0437	0.0710	0.0234	0.1504	0.0615	0.0396	0.3008	0.0269	0.0963
C46	0.0686	0.0626	0.0108	0.1710	0.0558	0.0540	0.2762	0.0299	0.1004
N51	0.0548	0.0819	0.0290	0.1747	0.0663	0.0545	0.2470	0.0141	0.0800
N52	0.0838	0.0690	0.0155	0.1928	0.0628	0.0548	0.2134	0.0158	0.0810
nitrate group A									
N6a	0.0880	0.0150	0.0230	0.1581	0.05148	0.0408	0.2630	0.0231	0.0791
O71a	0.0945	0.0397	0.0177	0.1572	0.01858	0.0482	0.1709	0.1042	0.0658
O71/1a	0.0963	0.0211	0.0190	0.1648	0.09338	0.0330	0.3113	0.1173	0.1056
O71/3a	0.0735	0.0413	0.0344	0.1544	0.04607	0.0424	0.3665	0.1125	0.0845
nitrate group B									
N6b	0.0335	0.0244	0.0127	0.1121	0.07239	0.0297	0.2356	0.0657	0.0709
O71b	0.0366	0.0284	0.0147	0.1357	0.0716	0.0294	0.2373	0.1405	0.0628
O71/1b	0.0240	0.0284	0.0156	0.0824	0.07718	0.0315	0.2966	0.1060	0.0767
O71/3b	0.0560	0.0342	0.0077	0.1197	0.07165	0.0294	0.2214	0.0829	0.0901
nitrate group C									
N8a	0.0605	0.0130	0.0283	0.2809	0.04792	0.0722	0.1739	0.0262	0.0502
O91a	0.1446	0.0718	0.0470	0.3138	0.04829	0.0897	0.4928	0.0456	0.1487
O91/1a	0.0268	0.0238	0.0409	0.2732	0.0405	0.0634	0.1911	0.0500	0.0583
O91/3a	0.0326	0.0639	0.0037	0.2604	0.08164	0.0645	0.5334	0.0056	0.0716

Table S3. Variation of distances and angles in the intermolecular N–H···O and C–H···O hydrogen bond motifs. Symmetry operations: (i) *x* – 1, *y*, *z*, *t*; (ii) *x* – ½, *y* + ½, *z*, *t*; (iii) –*x* + ½, –*y* + ½, *z* + ½, –*t* + ½; (iv) –*x*, –*y*, *z* + ½, –*t* + ½; (v) *x* + ½, *y* + ½, *z*, *t*; (vi) –*x* – ½, –*y* + ½, *z* + ½, –*t* + ½.

D–H···A	$d_{D–H}$ (Å)	$d_{H\cdots A}$ (Å)	$d_{D\cdots A}$ (Å)	∠(DHA) (°)
N21–H1n21···O71/1a^{i}	0.87	2.24 - 2.65	3.070(9) – 3.394(9)	144 - 165
N21–H1n21···O71/3a^{i}	0.87	2.48 - 2.67	3.137(9) - 3.279(9)	128 - 133
N22–H1n22···O71/1b	0.87	2.31 - 2.64	3.121(8) - 3.386(8)	144 - 155
N22–H1n22···O71/3b	0.87	2.43 - 2.60	3.107(8) - 3.256(8)	132 - 136
N23–H1n23···O71a	0.87	2.45 - 2.61	3.078(9) - 3.235(9)	128 - 136
N23–H1n23···O71/3a	0.87	2.27 - 2.46	3.088(9) - 3.243(9)	149 - 156
N24–H1n24···O71b^{i}	0.87	2.39 - 2.63	3.107(8) - 3.260(9)	130 - 138
N24–H1n24···O71/3b^{i}	0.87	2.32 - 2.42	3.139(9) - 3.229(9)	151 - 156
N25–H1n25···O71a^{ii}	0.87	2.34 - 2.72	3.138(9) - 3.440(9)	141 - 154
N25–H1n25···O71/1a^{ii}	0.87	2.42 - 2.65	3.076(9) - 3.279(9)	128 - 134
N26–H1n26···O71b^{ii}	0.87	2.25 - 2.39	3.078(8) - 3.200(8)	149 - 161
N26–H1n26···O71/1b^{ii}	0.87	2.45 - 2.57	3.089(8) - 3.231(8)	129 - 134
C31–H2c31···O71a^{ii}	0.96	2.29 - 2.42	3.201(10) - 3.371(10)	159 - 173
C32–H2c32···O71b^{ii}	0.96	2.15 - 2.25	3.062(10) - 3.173(10)	158 - 168
C33–H2c33···O71/1a^{i}	0.96	2.31 - 2.44	3.217(11) - 3.348(11)	156 - 171
C34–H2c34···O71/1b	0.96	2.22 - 2.37	3.150(10) - 3.328(11)	159 - 173
C35–H2c35···O71/3a	0.96	2.14 - 2.38	3.064(11) - 3.329(11)	161 - 171
C36–H2c36···O71/3b^{i}	0.96	2.26 - 2.36	3.164(10) - 3.274(10)	156 - 163
C41–H2c41···O91a^{ii}	0.96	2.19 - 2.43	3.149(10) - 3.320(10)	158 - 178
C42–H2c42···O91a^{iii}	0.96	2.38 - 2.66	3.332(10) - 3.618(9)	166 - 176
C43–H2c43···O91/3a	0.96	2.41 - 2.53	3.329(10) - 3.482(10)	161 - 178
C44–H2c44···O91/1a^{iv}	0.96	2.17 - 2.38	3.116(9) - 3.323(9)	168 - 173
C45–H2c45···O91/1a^{v}	0.96	2.27 - 2.46	3.227(11) - 3.393(11)	162 - 174
C46–H2c46···O91/3a^{vi}	0.96	2.25 - 2.50	3.185(11) - 3.453(11)	159 - 173

References

[1] V. Petricek, M. Dusek, L. Palatinus, *Z. Kristallogr*. **2014**, *229*, 345–352 and V. Petricek, M. Dusek, J. Plasil, *Z. Kristallogr*. **2016**, *231*, 583–599.

[2] S. Dey, A. Schönleber, S. Mondal, S. J. Prathapa, S. van Smaalen, F. K. Larsen, *Acta Crystallogr. B* **2016**, *72*, 372–380.

[3] A. Schönleber, S. Van Smaalen, F. K. Larsen, *Acta Crystallogr. C* **2010**, *66*, 107-109.

[4] V. Schomaker, K. N. Trueblood, *Acta Crystallogr. B* **1968**, *24*, 63–76.

[5] L. Li, A. Wölfel, A. Schönleber, S. Mondal, A. M. M. Schreurs, L. M. J. Kroon-Batenburg, S. van Smaalen, *Acta Crystallogr. B* **2011**, *67*, 205–217.
